# Comparison of the Fetomaternal Outcome in Women With Preterm Premature Rupture of Membranes on Expectant Management Versus Delivery at 34 Weeks

**DOI:** 10.7759/cureus.68917

**Published:** 2024-09-08

**Authors:** Shailja Jha, Purnima Saxena, Sumita Saluja, Harish Chellani, Jyotsna Suri, Bijoya Mukherjee, Sumitra Bachani

**Affiliations:** 1 Department of Obstetrics and Gynaecology, Vardhman Mahavir Medical College and Safdarjung Hospital, New Delhi, IND; 2 Department of Hematology, Vardhman Mahavir Medical College and Safdarjung Hospital, New Delhi, IND; 3 Department of Pediatrics, Vardhman Mahavir Medical College and Safdarjung Hospital, New Delhi, IND

**Keywords:** chorioamnionitis, management, prelabour rupture of membranes, preterm, sepsis

## Abstract

Context: This study aimed to study feto-maternal outcomes in women with preterm prelabor rupture of membranes (PTPROM) on expectant management versus delivery at 34 weeks of gestation and correlate the period of latency and inflammatory markers with delivery outcomes. We have chosen this research topic as there is a paucity of specific guidelines regarding the optimal period of gestation for delivering women with PTPROM.

Aim: The study correlated the feto-maternal outcomes in women with PTPROM on expectant management till 37 weeks versus delivery at 34 weeks with a period of latency and maternal inflammatory markers.

Methods and materials: This was a prospective observational study conducted on 262 women with PTPROM from 28-33+6 weeks of gestation. Women were monitored till 37 weeks with biweekly total leukocyte count and weekly C-reactive protein, urine routine microscopy, urine culture, high vaginal culture sensitivity, and ultrasound. Women were monitored expectantly till 37 weeks. However, intervention was done at any time during the feto-maternal compromise. There were 52 women who delivered <34 weeks and 210 women who delivered ≥34 weeks. Feto-maternal outcomes were documented. Group A was assigned to women who delivered before 34 weeks and Group B was assigned to women who delivered after 34 weeks. Statistical analysis was done using SPSS software. A p-value <0.05 was considered significant.

Results: Among the study group, 238 (90.8%) women were managed expectantly while 24(9.1%) required intervention. A latency of 3-4 weeks was observed in 131(50%) women. Chorioamnionitis developed in 7 women (4.4%) in group A and 13 women (4.9%) in group B. Neonates developed sepsis in 5.7% in group A and 5.8 % in group B and were comparable in both the groups (p=1.000). Early neonatal death (END) occurred in 10 (3.8%) among which seven died because of low birth weight (LBW), two due to sepsis, and one due to respiratory distress. LBW was significantly associated with END (p<0.001)

Conclusion: Expectant management beyond 34 weeks with close monitoring can improve neonatal outcomes without increasing maternal morbidity in women with PTPROM.

## Introduction

Preterm premature rupture of fetal membranes (PTPROM), a breach in the amniotic membranes prior to the onset of labor before 37 weeks of gestation, affects 3% of all pregnancies and results in 30-40% of preterm births [1]. Associated neonatal morbidity is primarily due to prematurity, sepsis, cord prolapse, and pulmonary hypoplasia [2,3].Serious maternal risks are chorioamnionitis and placental abruption. Improving neonatal outcomes without worsening maternal morbidity has been a challenge in such cases. It has been observed that increasing the period of latency from PTPROM to delivery can improve neonatal outcomes and reduce the morbidities of prematurity [3]. There are no specific guidelines about how long the latency period for optimal outcomes should be. American College of Obstetrics and Gynaecology (ACOG) recommends expectant management till 34 weeks of gestation but acknowledges that the recommendation is based on “limited and inconsistent scientific evidence” [1]. Royal College of Obstetrics and Gynaecology (RCOG) has updated that those who have no contraindication for continuing the pregnancy may be offered expectant management until 37 weeks with ongoing clinical assessment and discussing the risk and benefit ratio with the women [2]. The objective of the current study was to correlate the feto-maternal outcomes in women with PTPROM on expectant management till 37 weeks versus delivery at 34 weeks with period of latency and maternal inflammatory markers.

This study was previously posted to the Research Square preprint server in October 2023.

## Materials and methods

A prospective observational study of 262 women recruited from 28-33+6 weeks of gestation, who presented with PTPROM, was conducted for a period of 18 months from October 2020 to March 2022 in a tertiary care referral hospital in North India. The number of deliveries is around 2000/ month in the level 2 Neonatal intensive care unit (NICU).

Sample size

The study by Baser et al. observed that admission to the NICU was required in 25.24% at a gestation of 34 weeks [4]. Taking these values as a reference and assuming the difference of 15% in NICU admission between 34 and >34 weeks, the minimum required sample size with 90% power of study and 5% level of significance was 131 women in each study group. Considering the lost to follow-up as 5%, the total sample size taken was 300 (150 per group).

The formula used was n>=((pc*(1-pc)+pe*(1-pe))*(Zα + Zβ) 2 )/(pc-pe)2

pc: requirement of admission to the NICU in the gestational week of 34 weeks; pe: requirement of admission to the NICU in the gestational week of >34 weeks; Zα is the value of Z at a two-sided alpha error of 5%; Zβ is the value of Z at 90% power.

Singleton pregnancy with PTPROM was included in the study and women with chorioamnionitis, antepartum hemorrhage, severe preeclampsia and its complications, intrauterine death, and congenital malformations in fetus were excluded from the study.

**Figure 1:**
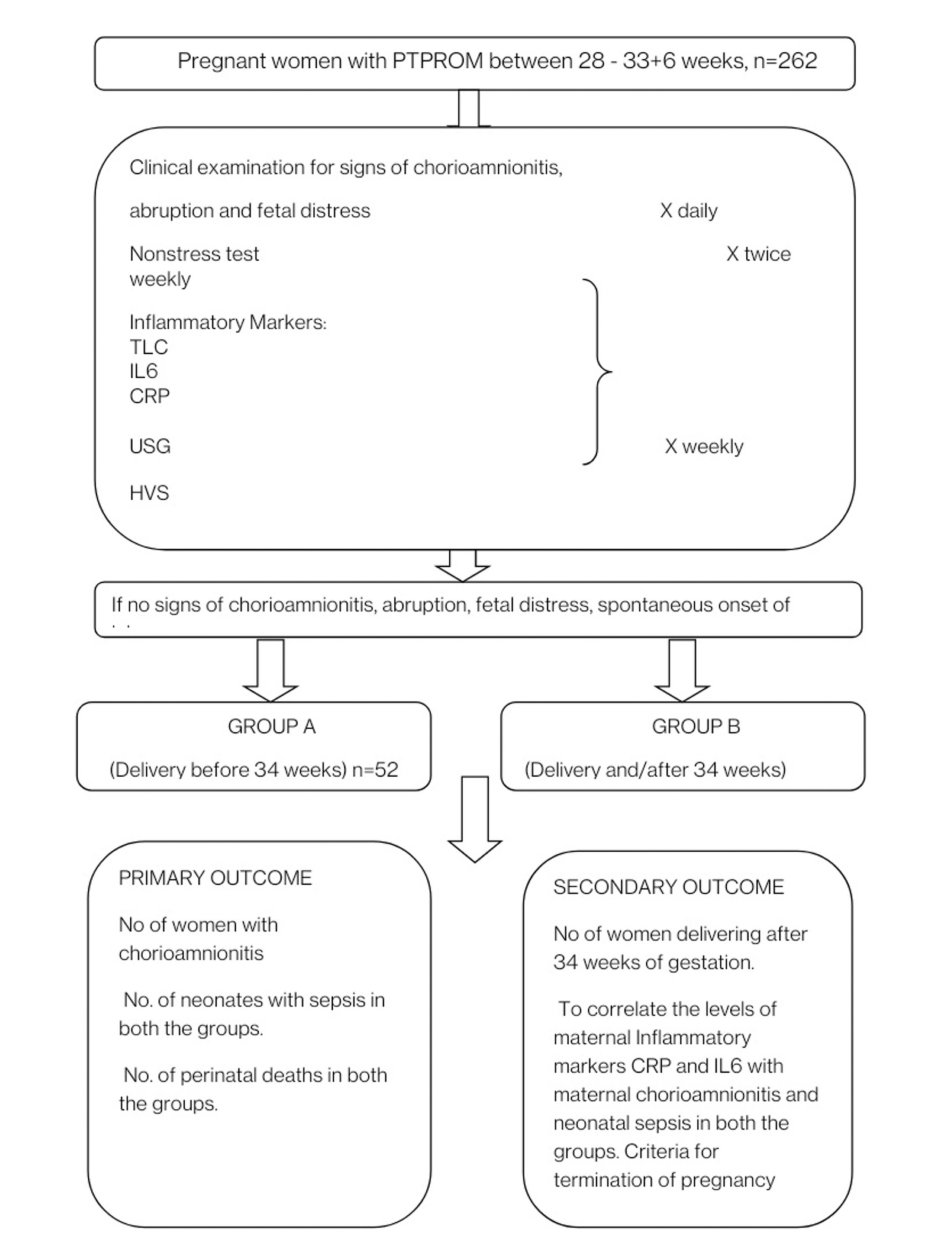
Flowchart depicting materials and methods Flowchart outlining the methods TLC: Total leukocyte count; USG: ultrasonography; HVS: high vaginal culture

The latency period was defined as the time interval from the onset of PTPROM to delivery. The study population was randomly allocated into two groups: those with delivery before 34 weeks (Group A) and those with expectant management at or beyond 34 weeks (Group B). Both groups underwent biweekly assessment of total leucocyte count (TLC), weekly urine, and high vaginal swab (HVS) culture. The ultrasonographic amniotic fluid index (AFI) was measured weekly and assessment of fetal wellbeing was done biweekly with non-stress test (NST) until delivery. C-reactive protein (CRP) and interleukin 6 (IL6) were measured weekly which were not included in the institutional protocol for termination of pregnancy and were blinded to the investigator in the study. Women were offered expectant management after informed consent and monitored till the 37-week period of gestation unless there was any indication for termination of pregnancy i.e. if the women developed chorioamnionitis, abruption, fetal distress, or spontaneous onset of labor as shown in Figure 1. Antibiotic prophylaxis of injection Ampicillin 1gram four times a day, injection Metronidazole 100mg three times a day were given for 2 days followed by oral Erythromycin 250 mg four times a day for seven days, and steroids were administered for fetal lung maturity. The outcome measures included the number of women with chorioamnionitis, the number of neonates with sepsis in both groups (delivering <34 weeks and delivery on/after 34 weeks), and the number of perinatal deaths in both groups. Secondary outcomes included the number of women delivering after 34 weeks of gestation and correlation of the levels of CRP and IL-6 with maternal chorioamnionitis and neonatal sepsis in both groups.

Ethics consideration

The study was performed after institutional ethical clearance, granted by the Ethics Committee of VMMC & Safdarjung Hospital (IEC/VMMC/SJH/Thesis/2020-11/CC-177).

Statistics

Normality of data was tested by the Kolmogorov-Smirnov test. Statistical tests were applied as follows: (i) Quantitative variables were compared using the unpaired t-test/Mann-Whitney Test (when the data sets were not normally distributed) between the two groups; (ii) Qualitative variables were compared using the Chi-Square test /Fisher’s exact test. Statistical analysis was done using IBM SPSS Statistics for Windows, Version 23 (Released 2015; IBM Corp., Armonk, New York, United States). A p-value <0.05 was considered significant.

## Results

Among 262 women, 52 women delivered at <34 weeks of gestation (group A), and 210 women delivered ≥34 weeks of gestation (group B). The demography was comparable in both the groups. In group A, 45(86.5%) and 7(13.4%) while in Group B 193(91.9%) and 17(8%) responded to expectant management and required intervention respectively (Table 1). Table 1 summarizes the maternal and fetal outcomes in both the groups. There was a total of 13 women who developed chorioamnionitis, 15 neonates developed neonatal sepsis, and 10 neonates developed early neonatal deaths. There was 9.6% chorioamnionitis in group A, whereas 3.8% in group B. Neonatal sepsis was seen in 5,8% in group A and 5.7% in group B which was comparable and early neonatal death was seen in 70% in group A and 30% in group B.

**Table 1:** Maternal and fetal outcome in Group A and Group B

Maternal Outcome	Group A n (%)	Group B n (%)
Expectant	45 (86.5)	193 (91.9)
Intervention	07 (13.4)	17 (8)
Spontaneous onset of labor	44 (84.6)	171 (81.4)
Vaginal delivery	50 (96.2)	202 (96.2)
Cesarean section	02 (3.8)	08 (3.8)
Abruption	03 (5.7)	03 (1.7)
Chorioamnionitis	5(9.6)	8(3.8)
Fetal Outcome		
NICU admission	21 (40.4)	36 (17.1)
Received surfactant	06 (11.1)	02 (1.0)
Neonatal sepsis	03 (5.8)	12 (5.7)
Early neonatal death	07 (70)	03 (30)

In group A, maximum women [34(65.4%)] had been recruited at 30-31+6 weeks, while in group B, maximum women [155(59.1%) were recruited at 32-33+6weeks.

In group A, 35(67.3%) women had a latency period of 1-2 weeks while 115(54.8%) women in group B had a latency period of 3-4 weeks. Amongst the total study group 131(50%) women had a latency of 3-4 weeks which was significant (p<0.001).

A total of 24(9.1%) women required intervention indicated for chorioamnionitis 13(4.9%), abruption 6(2.2%) and fetal distress 5(1.9%) (p-0.06). HVS culture was positive at delivery in 11.3% of women in the expectant group and 41.7% in the intervention group (Table 2).

**Table 2:** Delivery parameters (p-value has been calculated by Fisher’s exact test and Wilcoxon-Mann-Whitney U test)

Parameter	Group A (n = 52)	Group B (n = 210)	Total (N=262)	p-value
POG at admission	30.54±1.04	32.29±1.15		<0.001
mean ±SD(weeks)				
28-29+6Weeks	16(30.8%)	11(5.2%)	27(0.7%)	
30-31+6Weeks	34(65.4%)	46 (21.9%)	80 (30.5%)	
32-33+6Weeks	2(3.8%)	153(72.9%)	155(59.1%)	
Period of Latency				<0.001
<1Weeks	1(1.9%)	1(0.5%)	2(0.7%)	
1-2Weeks	35(67.3%)	73(34.8%)	108(41.2%)	
3-4Weeks	16(30.8%)	115(54.8%)	131(50%)	
5-7Weeks	0(0.0%)	19(9.0%)	19(7.3%)	
>7Weeks	0(0.0%)	2(1.0%)	2(0.8%)	
Indication Of Delivery				0.06
Abruption	3(5.7%)	3(1.7%)	6(2.2%)	
Chorioamnionitis	5(9.6%)	8(3.8%)	13(4.9%)	
Fetal distress	0(0.0%)	5(2.4%)	5(1.9%)	
Term	0(0%)	23(10.9%)	23(8.8%)	
Spontaneous onset of labor	44(84.6%)	171(81.4%)	215(82.1%)	
Birth weight (Kgs) Mean±SD	1.78±0.33	2.20±0.35		<0.001

In our study, 13(4.9%) women developed chorioamnionitis, 5(9.6%) in group A and 8(3.8%) in group B. Early preterm deliveries had a higher incidence of chorioamnionitis which was however not statistically significant (p=0.061). Amongst 13 women who developed chorioamnionitis, 6(50.0%) were recruited in the study at 32-33+6 weeks. There was no significant correlation between the period of gestation (POG) at admission and the development of chorioamnionitis. The majority of women 7(53.8%) developed chorioamnionitis at latency of 3-4 weeks and 6(46.1%) at latency of 1-2 weeks; however, this was not statistically significant. Inflammatory markers including raised TLC and CRP at admission and delivery (p<0.001) were significantly raised in women with chorioamnionitis. IL-6 levels had no correlation with the development of chorioamnionitis (Table 3).

**Table 3:** Analysis of women with chorioamnionitis in the study (p-value has been calculated by Fisher’s exact test and Wilcoxon-Mann-Whitney U test) TLC: Total leukocyte count; CRP: C-reactive protein; END: Early neonatal death; RDS: respiratory distress syndrome

Delivery parameters	Chorioamnionitis		p-value
	Yes(n=13)	No(n=249)	
POG at Delivery (Weeks) Mean ±SD	34.14±1.35	34.86±1.43	0.061
<34weeks (group A)	5(9.6%)	47(90.3%)	0.06
≥34weeks (group B)	8(3.8%)	202(96.1%)	
POG at admission			0.404
28-29+5 weeks	3(18.8%)	24(9.8%)	
30-31+6 weeks	4(31.2%)	76(30.5%)	
32-33+6 weeks	6(50.0%)	149(59.8%)	
Period of latency			0.829
<1 weeks	0 (0.0%)	2 (0.8%)	
1-2 weeks	7 (43.8%)	101 (41.1%)	
3-4 weeks	9 (56.2%)	122 (49.6%)	
5-7 weeks	0 (0.0%)	19 (7.7%)	
>7 weeks	0 (0.0%)	2 (0.8%)	
TLC (10³/dL) (admission)	12588.75 ± 2438.03	11406.50 ± 2342.21	0.077
TLC (10³/dL) (delivery)	15175.00 ± 4272.63	12659.92 ± 2181.81	0.011
CRP (admission)	8 (50.0%)	4 (1.6%)	<0.001
CRP (delivery)	7 (43.8%)	12 (4.9%)	<0.001
NICU admission	10(62.5%)	47(19.1%)	<0.001
Surfactant	3(18.8%)	5(2.0%)	0.009
Neonatal sepsis	3(23%)	12(3.7%)	<0.001
RDS	0(0.0%)	3(1.2%)	1
END	2(15.3%)	8(2.8%)	0.017

The mean birth weight in group A was 1.78±0.33 kgs and in group B was 2.20±0.35 kgs. Neonates in group B had increased birth weight likely due to advanced gestation (p<0.001). Among neonates, 15(5.7%) babies developed neonatal sepsis, 3(5.8%) in group A and 12(5.7%) in group B which was comparable(p=1.000). There was no correlation between the period of latency and development of neonatal sepsis. CRP as an inflammatory marker was observed to have significant association with sepsis both at admission (p=0.025) and delivery (p=0.016) (Table 4).

**Table 4:** Neonatal outcomes in the study group (p-value has been calculated by Fisher’s exact test and Wilcoxon-Mann-Whitney U test)

	Group A (<34 weeks) n=52	Group B (≥34 weeks) n=210	Total N=262	p-Value
Neonatal				
Outcomes				
Birth weight (Kgs) Mean±SD	1.78±0.33	2.20±0.35		<0.001
Neonatal sepsis	3 (5.8%)	12 (5.7%)	15 (5.7%)	1
NICU stay	21 (40.4%)	36 (17.1%)	57	<0.001
Duration of NICU stay (days)	1.12 ± 2.30	0.54 ± 1.67		0.032
Antibiotics	12 (23.1%)	8 (3.8%)	20	<0.001
Surfactant	6 (11.5%)	2 (1.0%)	8	<0.001

Among women whose babies had neonatal sepsis, the most common organism observed in HVS was streptococcus pneumoniae both at admission 2(13.3%) and at delivery 3(20.0%). HVS culture positivity at admission was not a significant parameter for neonatal sepsis; however at delivery, it was significantly associated with the development of chorioamnionitis (p=0.027). Among NICU admissions, 10(62.5%) among 13 were neonates of women with chorioamnionitis compared to 47(19.1%) amongst 249 neonates of other women (Table 3).

Most of the neonates, 12 (80%) with neonatal sepsis delivered at <34 weeks, and their average weight was 1.84±0.42kgs. In group A, 21(40.4%) babies were admitted to the NICU likely due to early preterm delivery, while in group B, 36(17.1%) babies were admitted in NICU. More babies in group A 12(23.1%) received antibiotics than in group B 8(3.8%) which was significant (p<0.001). Neonatal sepsis was diagnosed in 3(23%) newborns whose mothers had chorioamnionitis and 2(15.3%) amongst these had early neonatal death but it was not significant (Table 5).

**Table 5:** Relation of neonatal sepsis in the study (p-value has been calculated by Fisher’s exact test and Wilcoxon-Mann-Whitney U test) RDS: Respiratory distress syndrome; END: early neonatal death

	Neonatal sepsis		p-value
	Yes(n =15)	No(n =247)	
PTROM-Presentation Interval (hours)mean±SD	50.27±38.86	35.53±35.14	0.063
Period of Latency (weeks) mean±SD	2.60±1.68	2.90±1.33	0.262
<1Weeks	0(0.0%)	2(0.8%)	
1-2Weeks	8(53.3%)	100(40.5%)	
3-4Weeks	6(40.0%)	125(50.6%)	
5-7Weeks	1(6.7%)	18(7.3%)	
>7Weeks	0(0.0%)	2(0.8%)	
Chorioamnionitis	3(20%)	10(4%)	
Spontaneous onset of labor	8(53.33%)	208(84.2%)	
Birth weight (Kg) mean±SD	1.84±0.42	2.14±0.37	0.015
NICU admission	15(100.0%)	42(17.0%)	<0.001
NICU stay	3.93±3.17	0.46±1.51	<0.001
Antibiotics	15(100%)	13(5.3%)	<0.001
Surfactant	1(6.7%)	7(2.8%)	0.38
RDS	0(0.0%)	1(1.2%)	1
END	2(13.3%)	8(3.2%)	0.106

Early deaths occurred in occurred in 10 neonates among which 7(70%) died because of low birth weight (LBW), 2(20%) due to neonatal sepsis, and 1(10%) due to respiratory distress. Among these, 70% had been delivered before 34 weeks. LBW was significantly associated with early neonatal deaths (p<0.001) (Table 6).

**Table 6:** Neonatal outcome in relation to early neonatal death ( p-value has been calculated by Fisher’s exact test and Wilcoxon-Mann-Whitney U test) RDS: Respiratory distress syndrome

Parameters	Early Neonatal Death		p-value
	Yes(n==10)	No(n=252)	
Birth weight (Kg)	1.38 ± 0.31	2.15 ± 0.35	<0.001
NICU admission	9 (90%)	47 (19.0%)	<0.001
NICU stay	2.30 ± 2.50	0.59 ± 1.77	<0.001
Antibiotics	8 (80.0%)	12 (4.8%)	<0.001
Surfactant	5 (50.0%)	3 (1.2%)	<0.001
Neonatal sepsis	2 (20.0%)	13 (5.2%)	0.106
Low birth weight	7 (70%)	0 (0%)	<0.001
RDS	1 (10.0%)	2 (0.4%)	0.004

## Discussion

Our study highlights the advantages of optimizing the pregnancy outcomes in PTPROM and delivery beyond 34 weeks. In a study by Drassinower et al. comprising 1596 women with a latency period of one week in 25.4%, it was concluded that prolonging the latency in PTPROM was associated with decreased incidence of neonatal sepsis and delivery at earlier gestational age was associated with the adverse neonatal outcome [5]. Minakeshi et al. observed that the mean period of latency was inversely proportional to gestation at rupture of membranes. They observed women who came at lower gestational age had longer periods of latency [6]. Lorthe et al studied 702 women with PTPROM at 24-32 weeks with a latency of 3-7 days in 38% of women and reported that prolonging the latency increased the survival rates with a decrease in neonatal morbidities [7]. Baser et al. reported favorable feto-maternal outcomes in a study on 206 women between 24-34+6 weeks with a mean latency period of 15.1±13.8days [4]. These data were similar to our study wherein 50% of women in the study group had a latency of 3-4 weeks on expectant management.

In a multicenter study by Lynch et al., an increased incidence of chorioamnionitis in the expectant group until 35 weeks was noted as compared to immediate delivery at 34 weeks [8]. This was in contrast to our study, and the difference could be attributed to the difference in the gestational age of delivery of the study groups. van der Ham et al. conducted the PPROMEXIL metanalysis on 195 women with PTPROM between 34 and 37 weeks to study whether induction of labor reduced neonatal sepsis. Similar to the study they observed a comparable number of cesarean sections in both the immediate delivery and expectant management group [9]. The Cochrane review conducted on 3617 women between 26-37 weeks observed a higher incidence of chorioamnionitis in the active management group than in the expectant group [3]. In a study by Kartikeshwar et al. comprising 197 women, clinical chorioamnionitis was detected in 4.7% of the expectant management group only though was not statistically significant [10]. There was no correlation between the latency period and the onset of chorioamnionitis in our study.

Stepan et al. reported that the 95th centile of CRP in PTPROM had a very low sensitivity of 15% and, a positive predictive value of 90% (with a false positive rate of 1%), thereby rendering it clinically a poor predictor of microbial invasion of the amniotic cavity and histological chorioamnionitis [11]. A meta-analysis by Etyang et al. reported that there was insufficient evidence to prove that inflammatory markers such as CRP, IL6, and procalcitonin as a solitary test for the diagnosis of chorioamnionitis and marker for taking decision of termination of pregnancy in PTPROM [12].

Morris et al. reported an incidence of 2% neonatal sepsis in the immediate delivery group and 3% in the expectant, which was comparable to our study. They concluded that expectant management should be adopted as a plan of management in PTPROM women without any other high-risk factor and termination of pregnancy only in maternal signs of infection or fetal distress [13].

In a meta-analysis by Quist-Nelson et al., no difference in neonatal morbidity and mortality in women in the expectant or active management group was reported [14], which was similar to our study as most neonatal complications were due to low birth weight and prematurity.

The limitation of the study was that it was not a randomized study; hence power of the study was less. Both study groups were not equally distributed.

## Conclusions

Women with PTPROM can be offered expectant management beyond 34 weeks of gestation as latency of 3-4 weeks is not significantly associated with chorioamnionitis. Rising TLC and positive CRP are indicators for delivery in these women. The incidence of neonatal sepsis is higher in women with raised CRP at admission and delivery. Expectant management with close monitoring can improve neonatal outcomes without increasing maternal morbidity in PTPROM.
